# Acknowledgement to Reviewers of *Bioengineering* in 2018

**DOI:** 10.3390/bioengineering6010006

**Published:** 2019-01-14

**Authors:** Bioengineering Editorial Office

**Affiliations:** MDPI, St. Alban-Anlage 66, 4052 Basel, Switzerland

Rigorous peer-review is the corner-stone of high-quality academic publishing. The editorial team led by Prof. Anthony Guiseppi-Elie, Editor-in-Chief, and Associate Editors, Dr. Mark Blenner, Prof. Liang Luo and Dr. Rossana Madrid greatly appreciate the reviewers who contributed their knowledge and expertise to the journal’s editorial process over the past 12 months. In 2018, a total of 108 papers were published in the journal, with a median time to first decision of 15.5 days and a median time to publication of 42 days. The editors would like to express their sincere gratitude to the following reviewers for their cooperation and dedication in 2018:

Abouhossein, AlirezaMaltesen, RalucaAcevedo, Rubén CarlosMänner, JörgAchinivu, EzinneMarcal, HelderAhmed, SanaMariscal, VicenteAkhlaghi, Seyedeh ParinazMarszalek, KrystianAltvater, MartinMassai, DianaAmerinatanzi, AmirhesamMcHugh, KevinAn, ShuaiMehedi, MasfiqueArany, PraveenMehrabi, RezaArunkumar, AbhiramMelvin, AdamAzizian Kalkhoran, MohammadMerrin, JackBachmann, HerwigMicheli, LauraBai, JingMilcovich, GesmiBallesté, Ruben MasMin, LeeBanerjee, UddyalokMitra, KunalBaran, JaroslawMitra, Siddhartha ShankarBarba, Francisco J.Moazen, MehranBarbeck, MikeModaresi, SamanBarbedo, Jayme Garcia ArnalMondal, SudipBarrios Muriel, JorgeMotohashi, KenBartsch, RonnyMutreja, IshaBeebe, StephenNaik, Ganesh RBiedermann, ThomasNanjundaswamy, AnandaBiswas, AniketNaoki, KuritaBiswas, DipankarNawaz, MuhammadBlaser, MarkNeubauer, PeterBrisk, PhilipNguyen, TrieuBurger, MichaelNguyen, Trong-NguyenBuzdin, AntonNi, DalongCapizzi, GiacomoNivedita, NiveditaCarriel, VíctorNoort, Danny VanCausa, FilippoNurunnabi, MdChaudhuri, Parthiv KantOpdam, NiekChen, Chi-HuaOrbach, Sophia M.Chen, Han-Chiao IsaacOsaki, TatsuyaChen, LinOsanai, TakashiChen, WenchunPacelli, SettimioCho, Hea-YoungPapantonopoulos, GeorgeCho, Ssang-GooPeterson, CatherineChoudhuri, AvikPetrosanu, Dana-MihaelaCicone, FrancescoPhilips, NeenaCoburn, JeanninePhoenix, TimothyContessi Negrini, NicolaPineault, NicolasCourtial, Edwin-JofreyPinho, DianaCousins, FionaPistelli, FrancescoCredi, CaterinaPoggio, ClaudioCuviello, AndreaPradhan, KamneshDash, BirajaPradhan, ShantanuDash, RanjeetPrados-Frutos, Juan-CarlosDawn, ArnabPrados-Privado, MaríaDe Candia, PaolaPrince, Roger C.Deska, JanQiu, HaoDijkstra, Albert J.Rahman, MunsurDombrowski, StephenRai, PrakashDöppner, Thorsten R.Raina, MedhaDuarte Campos, Daniela FilipaRampazzo, ElenaDuisit, JeromeRanc, VáclavDurand, MarlèneRanzato, EliaEbramzadeh, EddieRao, SmithaEisenstat, David D.Raposo, MariaEiteman, MarkRavuri, SudheerElsaadany, MostafaRebelo, Sofia P.Eschbaumer, MichaelRemesh, SoumyaEsfandyarpour, RahimRen, KaixuanEttayapuram Ramaprasad, Azhagiya SingamRen, XiFare, SilviaRho, Hoon SukFarshbaf, SepidehRoche, EllenFaustino, AnaRödel, FranzFissell, WilliamRojo, Maria AngelesFocaroli, StefanoRojo-Álvarez, José LuisFountain, Augustus W.Romanowicz, PawelFuhrmann, GregorRoscini, LucaGajjar, ChiragRotermund, DavidGao, YunxiangSaccomandi, PaolaGarofalo, MariangelaSalerno, SimonaGiacomozzi, ClaudiaSanz Garcia, AndresGlowacz, AdamSapudom, JiranuwatGómez-Ruiz, SantiagoSchmitt, SynGonzalez-Gutierrez, JoaminSerrano, Dolores R.Gouws, ChrisnaSesay, Adama MarieGowrishankar, Thiruvallur R.Shah, Furqan A.Greetham, DarrenShelke, GaneshGuan, WenjianShi, YubingGurramkonda, ChandrasekharShornick, LaurieHadjiiski, LubomirShukuya, MasanoriHajba, LaszloSimutis, RimvydasHalpenny, Genevieve M.Singh, YogeshHamilton, NickSinyukov, AlexanderHamon, MorganSkretas, GeorgiosHao, JinsongSoh, Jun HuiHast, MichaelSolitro, GiovanniHautefeuille, MathieuSong, ChangsikHendricks, MarkSperling, Laura ElenaHmaidouch, RimSpolaor, FabiolaHossain, Md ShakhawathSredni, Simone TreigerHristov, IlianSridhar, KaushikHu, Michael SSriram, GopuHua, JiaSteinbach-Rankins, JillHuh, Yong-MinStrauss, SarahHuling, JenniferStruk, PrzemyslawIbarlucea, BergoiSubramaniyan, ManivannanIbrahim, HamdySuk, Jung SooJaberolansar, NoushinSun, ChenJackman, Jane E.Tan, ZaigaoJankovic, MomciloTaunk, Neil KJha, MithileshTerpou, AntoniaJin, KailongThiruvengadam, MuthuJohnson, JamesThompson, Karl ManleyJoseph, SusanThrockmorton, AmyKaczmarek, MariuszTimmers, LeoKaizer, Marina Da RosaTorlapati, JagadishKamaleswaran, RishikesanTreglia, GiorgioKang, MingonTsai, Ang-ChenKantak, ChaitanyaTsurui, HiromichiKaraś, MagdalenaTurner, NeillKarasiński, JanuszUccheddu, FrancescaKashaninejad, NavidValentini, FrançoiseKavanagh, JohnVarache, MathieuKehr, Nermin SedaVarcoe, Ramon L.Khan, IrfanVarghese Chacko, JenuKhan, MohsinVasudevan, Ananda Ayyappan JaguvaKhurshid, ZohaibVaz, JosianaKim, JanghoVenkat, VangavetiKirillova, AlinaVenkatesan, JayachandranKlar, AgnesViefhues, MartinaKlosterhalfen, BerndVolpe, MaurizioKochanowski, KarlVon Langermann, JanKoizumi, MitsuruVon Lieres, EricKoller, MartinWandzik, IlonaKowtharapu, Bhavani S.Wang, BoKrautwald, StefanWang, DepengKrištof, KolomanWang, JiongweiKrólicka, AleksandraWang, KaiKrstanović, VinkoWang, LeyiKucinska-Lipka, JustynaWang, QinyiKundu, BijoyWang, ShengKung, EthanWang, YifeiKusuma, GinaWayne, Jennifer S.Kwon, YongchanWeihs, DaphneLake, Spencer P.Wong, Siu LingLang, IngridWood, David W.Lanterna, AnabelWöstemeyer, JohannesLanza, GMWright, CatherineLaprie, AnneWu, JiandongLavandera, IvánXu, TaoLeung, Hui MinYan, JipengLi, XiangYang, GuangLi, XueYang, JingLi, ZidaYang, Yueh-Hsun KevinLiao, ChenYaniv, YaelLipiński, SewerynYaqub, MaqsoodLiu, YongliangYost, Michael J.Lowell, AndrewYu, Hoi-Ying ElsieLuo, XiaozhouZambon, AlessandroLyle, Alicia N.Zeilinger, KatrinMa, ChaoZhang, BoyangMaeda, YusukeZhao, WenxiuMajtan, JurajZhen, XuMajumder, PoulamiZiegler, DavidMalliaras, KonstantinosZopf, DavidMallis, Panagiotis


